# Proliferating Cell Nuclear Antigen (PCNA) Interactions in Solution Studied by NMR

**DOI:** 10.1371/journal.pone.0048390

**Published:** 2012-11-06

**Authors:** Alfredo De Biasio, Ramón Campos-Olivas, Ricardo Sánchez, Jorge P. López-Alonso, David Pantoja-Uceda, Nekane Merino, Maider Villate, Jose M. Martin-Garcia, Francisco Castillo, Irene Luque, Francisco J. Blanco

**Affiliations:** 1 Structural Biology Unit, CIC bioGUNE, Derio, Spain; 2 Spectroscopy and NMR Unit, Centro Nacional de Investigaciones Oncológicas, Madrid, Spain; 3 Instituto de Química Física Rocasolano, Consejo Superior de Investigaciones Científicas, Madrid, Spain; 4 Department of Physical Chemistry and Institute of Biotechnology, University of Granada, Granada, Spain; 5 IKERBASQUE, Basque Foundation for Science, Bilbao, Spain; National Institute for Medical Research, Medical Research Council, United Kingdom

## Abstract

PCNA is an essential factor for DNA replication and repair. It forms a ring shaped structure of 86 kDa by the symmetric association of three identical protomers. The ring encircles the DNA and acts as a docking platform for other proteins, most of them containing the PCNA Interaction Protein sequence (PIP-box). We have used NMR to characterize the interactions of PCNA with several other proteins and fragments in solution. The binding of the PIP-box peptide of the cell cycle inhibitor p21 to PCNA is consistent with the crystal structure of the complex. A shorter p21 peptide binds with reduced affinity but retains most of the molecular recognition determinants. However the binding of the corresponding peptide of the tumor suppressor ING1 is extremely weak, indicating that slight deviations from the consensus PIP-box sequence dramatically reduce the affinity for PCNA, in contrast with a proposed less stringent PIP-box sequence requirement. We could not detect any binding between PCNA and the MCL-1 or the CDK2 protein, reported to interact with PCNA in biochemical assays. This suggests that they do not bind directly to PCNA, or they do but very weakly, with additional unidentified factors stabilizing the interactions in the cell. Backbone dynamics measurements show three PCNA regions with high relative flexibility, including the interdomain connector loop (IDCL) and the C-terminus, both of them involved in the interaction with the PIP-box. Our work provides the basis for high resolution studies of direct ligand binding to PCNA in solution.

## Introduction

**Figure 1:**
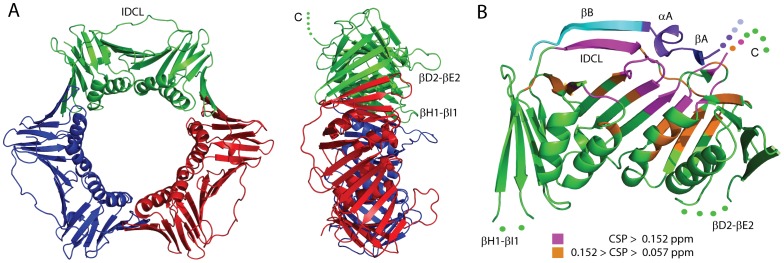
Structure of PCNA free and bound to p21 PIP-box peptides. (**A**) Ribbon scheme of the crystal structure of human PCNA trimeric ring (PDB entry 1VYM) front and side views showing the three protomers in different colors. The IDCL (residues 117–134), the βD2−βE2 loop (184–195), the βH1−βI1 (residues 105–109) and the C-terminus are labeled on the protomer colored in green. The five C-terminal residues (residues 256–261) not seen in the crystal structure are indicated by circles in the same protomer. (**B**) Ribbon representation of the crystal structure of one of the PCNA protomers bound to the p21^22^ peptide (PDB entry 1AXC) and the CSPs caused by p21^20^ peptide binding in solution. The short αA helix of the p21^22^ peptide docks into a hydrophobic pocket partly formed by the C-terminal half of the IDCL of PCNA, while the peptide βB strand interacts mostly with the extended N-terminal half of the IDCL. The residues not seen in the crystal are indicated by circles. The p21 residues in common with both the p21^12^ and p21^20^ peptides are colored in purple, the ones in common only with the p21^20^ peptide in cyan, and the two N-terminal residues not present in any of these two peptides are colored in light blue. The labels indicate the IDCL, two loops with missing residues in the crystal and the C-terminus of PCNA and the secondary structural elements of the p21^22^ peptide. Those PCNA residues that experience CSPs larger than the average (0.057 ppm), or larger than the average plus one standard deviation (0.152 ppm), are colored in orange and in magenta, respectively. Except for the IDCL, the loops are labeled using the original nomenclature, indicating with a greek letter the types of secondary structure elements connected by the loop, with a capital latin letter the order of those elements along the PCNA sequence, and with a number the corresponding pseudodomain of the PCNA protomer [14]. This figure was prepared with the program PyMol (Schrödinger).

DNA sliding clamps are central components of the DNA replication machinery. They consist of multimeric, toroidal-shaped structures with pseudo-six fold symmetry that encircle the DNA duplex and act as processivity factors during replication by tethering the polymerases to the genomic template. All kingdoms of life retain functionally and structurally related sliding clamps that differ in the multimeric association of monomeric subunits [1]. The bacterial clamp (DNA polymerase III β subunit) is formed by the homo-dimeric association of two protomers, each one with three topologically similar domains [2], [3]. In contrast, the archaeal and eukaryotic clamps assemble into trimeric rings in which each protomer contains two similar domains and a long interdomain-connecting loop (IDCL), as illustrated in Figure 1A [3], [4]. The PCNA protomers are arranged in a head-to-tail fashion forming a ring with two distinct faces: one with prominent loops that protrude into the solvent, and another with the three IDCLs linking the two domains of each protomer and the C-termini of the three chains, known as the C-side (Figure 1A). The PCNA rings are stable in solution [5] and need to be opened to be loaded onto the DNA [6]. The clamp loader (replication factor C, RFC) mediates the assembly of PCNA onto DNA in an ATP dependent process [7].

In addition to the replicative function, PCNA directs other important cellular processes through the interaction with a host of DNA-processing proteins and cell cycle regulators [8]. Many of the proteins that interact with PCNA contain a conserved sequence known as PIP-box (PCNA Interacting Protein-box). The pattern of the PIP-box sequence is *QXXhXXaa*, where *h* is an aliphatic hydrophobic residue, *a* is an aromatic hydrophobic one (typically F or Y), and *X* is any of the 20 proteinogenic amino acids [9].

The crystal structure of Flap endonuclease 1 (Fen-1) bound to human PCNA is the only structure available of a full length protein bound to PCNA [10]. It shows one Fen-1 molecule bound to each one of the three PCNA protomers. The core domain of Fen-1 interacts with some PCNA loops and with its C-terminus, but the largest interface is formed by the C-terminal tail of Fen-1, which contains a PIP-box that sits into a channel on the surface of PCNA. This tail is folded into a short β-strand (βA), a one-turn helix (αA), and a long β-strand (βB). The βA and βB strands form antiparallel β-sheets with PCNA regions at the C-terminal end and the IDCL, respectively. The face of the helix containing the conserved hydrophobic residues of the PIP-box docks into a hydrophobic pocket of PCNA. The three Fen-1 molecules do not interact with each other, suggesting independent binding events, and their active sites are oriented so that they have no access to the DNA duplex. It is thought that Fen-1 can switch its core domain between a locked inactive orientation to a tethered complex capable of a productive interaction with the DNA, a switch made possible by the hinge region between the core domain and the C-terminal segment. The crystal structure of the *Archaeoglobus fulgidus* RNaseHII/PCNA complex also shows three unique orientations as the enzyme rotates about a flexible hinge while anchored to each PCNA protomer by its PIP-box [11]. Flexibility in the PIP-box may be a common feature of proteins that bind PCNA through this sequence [12].

There are crystal structures of human PCNA bound to PIP-box peptides from four different proteins: Fen-1, the Cyclin-dependent kinase inhibitor 1 (CDKN1A, also known as p21^WAF1/CIP1^, and hereafter referred to as p21), the subunit 3 of the human replicative DNA polymerase-δ (POLD3, also known, and hereafter referred to as p66), and the B subunit of RNaseH2 (RNaseH2B) [11], [13], [14]. The crystal structure of the 22-residue long p21 139–160 fragment (p21^22^) bound to PCNA (Figure 1B) shows the peptide folded into a short N-terminal βA strand interacting with the PCNA C-terminus, a one turn 3_10_ helix αA in a hydrophobic pocket (including the C-terminal half of the IDCL), and a C-terminal βB strand interacting with the N-terminal half of the IDCL [14]. This structure is essentially the same as the C-terminal end of the Fen-1 protein bound to PCNA. The other peptides bound to PCNA adopt conformations very similar to the p21 peptide but with shorter βB-strands. For instance, only 11 residues are visible in the structure of the 20-residue long Fen-1 331–350 fragment (Fen-1^20^) bound to PCNA [13].

Our previous assignment of the NMR resonances of PCNA [15] allows to examine ligand binding in solution by measuring the chemical shift perturbations (CSP) of the PCNA resonances. As a benchmark for NMR studies on PCNA ligand interactions we have characterized the binding of the PIP-box peptide of p21. Our measurements in solution are consistent with the crystal structure of the complex. However, we have observed very weak or no detectable binding when studying the PIP-box fragment of the tumor suppressor ING1, the Cyclin-dependent kinase 2 (CDK2) protein, or the C-terminal domain of Myeloid cell leukemia 1 (MCL-1) proteins, which have been reported to interact with PCNA [16], [17], [18]. The NMR analysis of the backbone dynamics of PCNA in the ns-ps time scale, also presented here, shows that some of the regions involved in PIP-box binding are highly flexible relative to other parts of PCNA in solution.

## Materials and Methods

### Protein Expression and Purification

Human PCNA protein (UniProt entry P12004) was produced and purified as previously described [15]. Briefly, the genes were subcloned with an N-terminal His_6_ tag and a PreScission protease cleavage site. Protein samples with natural isotopic abundance or uniformly enriched in ^2^H,^15^N or in ^2^H,^13^C,^15^N were obtained by expression in *E. coli* cells grown in the appropriate culture media. The proteins were purified from the soluble fraction by several chromatography steps, and contained the extra sequence GlySerHis- at the N-terminus after proteolysis and removal of the affinity tag. Stock solutions in PBS (137 mM NaCl, 2.7 mM KCl, 10 mM sodium phosphate, 2 mM potassium phosphate) adjusted to pH 7.0 with HCl were flash frozen in liquid nitrogen and stored at −80°C. The pH of PBS was adjusted to pH 7.0 instead of pH 7.4 to improve the sensitivity of NMR measurement of the amide protons. Protein concentration of the samples was measured by absorbance at 280 nm using the extinction coefficient calculated from the amino acid composition. The concentrations of PCNA samples indicated in this work always refer to the corresponding concentration of protomers. Mass spectrometry analysis confirmed the integrity of the purified polypeptide chain and indicated a very high level of isotope enrichment in the different samples.

The human MCL-1 ΔN151−ΔC7 construct cloned in vector pET32a was expressed and purified as described [19]. Briefly, the protein was expressed in *E. coli* BL21(DE3) cells with a histidine-tag and a Tev protease site at the N-terminus, and was purified by two chromatography steps: immobilized nickel ion affinity and size exclusion.

Human CDK2 was expressed and purified as described [20]. Briefly, the protein was expressed in *E. coli* BL21(DE3) cells with a His_6_-SUMO-1 N-terminal tag which, after immobilized metal affinity chromatography, was removed by the Senp2 protease.

### NMR Measurements and Analysis

Unless indicated otherwise, the NMR spectra were recorded on a Bruker AV600 spectrometer equipped with a cryogenically-cooled triple resonance z-gradient probe at 35°C on PCNA samples with or without ligands in PBS pH 7.0. The spectra in Figures S2, S4, S7 and S8 were recorded on a Bruker AV800 spectrometer with a cryogenically-cooled triple resonance z-gradient probe on samples and buffers described in the corresponding legends. TROSY-based 3D HNCO, HN(CA)CO, HNCACB, HN(CO)CACB, and HNCA [21] were recorded for resonance assignment. Spectra were processed with TopSpin (Bruker) or with NMRPipe [22] and analyzed using NMRView [23]. Peak intensities were evaluated by the peak heights measured using CCPNMR [24].

^15^N relaxation data were collected at 60 MHz on a 1 mM PCNA sample. The ^15^N-{^1^H} heteronuclear NOE were measured in the interleave mode with an overall recycling delay of 10 s to ensure the maximal development of NOEs before acquisition and to allow solvent relaxation, thus avoiding transfer of saturation to the most exposed amide protons of the protein between scans [25]. For the T_2_ measurements 9 different spectra were recorded with transverse relaxation times of 6, 10, 14, 22, 26, 30, 38, 42, and 62 ms. The T_1_ values were obtained from spectra recorded at longitudinal relaxation delays of 7, 505, 1003, 1506, 2204, 3005, and 4001 ms. All spectra were recorded with TROSY versions of the corresponding experiments [26]. Most of the measured ^15^N-{^1^H} NOEs fall within the theoretical limits (maximum 0.82, minimum −3.5) of this parameter at 60 MHz, indicating that systematic errors or artifacts, if any are small, and, therefore, that the measurements are reliable. Relaxation times were considered reliable after visual inspection of the fitting curves and when the RMSD of the fitting was small. The global analysis of the relaxation parameters was done essentially as described [27].

For the assignment of the NMR spectrum of PCNA bound to p21^12^, a sample of ^2^H,^13^C,^15^N-PCNA was titrated with aliquots of a 13 mM stock of p21^12^ adding 0.1 equivalents of peptide up to 1.5 equivalents, and acquiring a ^1^H-^15^N correlation spectrum after each addition (Figure S5). At the end of the titration the sample was 0.9 mM/1.4 mM (PCNA/p21^12^) and a series of triple resonance spectra were acquired for sequence specific assignment. The same strategy was not possible with the complex with p21^20^ because of its low solubility. A sample containing 0.5 mM/0.6 mM (^2^H, ^13^C, ^15^N-PCNA/p21^20^) but partially precipitated did not yield useful triple resonance spectra. The assignment of the ^1^H-^15^N correlation spectrum of this sample was made by comparison with the assigned spectrum of the complex with p21^12^ and the shifts observed in the titration series with this peptide. The assignments of human PCNA bound to p21^20^ and p21^12^ peptides have been deposited in the Biological Magnetic Resonance Data Bank with entry numbers 17375 and 17376, respectively.

The values of the CSPs were computed with the equation: CSP = sqrt(((Δδ_H_)^2^+(Δδ_N_/5)^2^)*0.5), where Δδ_H_ and Δδ_N_ are the chemical shift changes in the ^1^H and ^15^N resonances, respectively, upon peptide addition. An experimental error of 0.03 pm in the CSP values can be computed in a similar way from the digital resolution in the corresponding spectral dimensions of the spectrum [28]. However this error is overestimated as evidenced by the much smaller CSP values for the PCNA residues in the presence of a ligand that does not bind or binds very weakly, such as the ING^22^ peptide. The average of the CSPs measured in the presence of this peptide was 0.005 ppm, providing a more realistic estimation of the uncertainty in the CSP values. The dissociation constant (*K_d_*) of the PCNA/ING1^22^ peptide was determined from a titration of a 280 µM sample of U-^2^H,^15^N-PCNA with increasing amounts of a 7.5 mM stock of the peptide. The data were fitted (using Origin, Microcal) to the equation: CSP = (K_d_+[P]+[I]–sqrt((K_d_+[P]+[I])^2^−4*[P]*[I]))/(2*[P])*CSP_max_, where [I] is the concentration of the ING1^22^ peptide at each titration point, [P] is the concentration of PCNA, and CSP_max_ is the CSP when PCNA is saturated, at infinite peptide concentration. CSP and [I] are the experimental values at each titration point, and [P] is the initial concentration of PCNA, assumed to be constant during the titration. CSP_max_ was either treated as a free adjustable parameter in the fitting (together with *K_d_*), or set as a constant equal to the value measured in the PCNA/p21^20^ complex, as described in the text.

### Isothermal Titration Calorimetry

ITC experiments were performed using a high-precision VP-ITC titration calorimetric system (Microcal Inc., Northampton, MA). The PCNA samples were extensively dialyzed against the titration buffer. The lyophilized peptides were dissolved in the dialysis buffer and the pH adjusted with NaOH and HCl if necessary. All solutions were filtered, properly degassed to avoid bubble formation, and equilibrated to the desired temperature prior to each experiment. The protein solution (at concentrations in the range 11.2–13.4 µM) in the calorimetric cell was titrated with the appropriate peptide ligand (at 117 µM for p2120 and 363 for p2112). A constant injection volume of 12 µL was used for the p2120 peptide. In the case of the shorter p2112 peptide, due to its lower binding affinity and to define the titration curve, a profile of injection volume ranging from 4 to 21 µL was used. The heat evolved after each peptide injection was obtained from the integral of the calorimetric signal. The resulting binding isotherms were analyzed by non-linear least-square fittings of the experimental data to a model corresponding to a single set of identical sites, as previously described [29]. Data analysis was done with Microcal Origin (OriginLab) together with software developed in our laboratory.

## Results

### The IDCL and the C-terminal Regions of the PCNA Backbone are Highly Dynamic in Solution

NMR provides exclusive information on the internal dynamics of the protein backbone through ^15^N spin relaxation measurements. For this purpose we have measured the ^15^N T_1_ and T_2_ relaxation times, and the ^15^N-{^1^H} heteronuclear NOE at 35°C on the PCNA ring (Figure S1). Despite the NMR technical difficulties associated to a system of this size (87 and 98 kDa in the natural abundance and triply labeled forms, respectively), good quality spectra were obtained with perdeuterated samples of PCNA at 1 mM concentration. This allowed the proper quantification of the intensities that, in turn, yielded precise values for the relaxation times by non-linear fit to decaying exponentials and for the ^15^N-{^1^H} NOE by calculating the ratio of intensities in spectra with and without saturation of the amide protons.

The relaxation parameters could be reliably measured for more than two thirds of the backbone amides. The remaining amide signals presented excessive overlap with other resonances or intensities too low for an accurate quantification. The magnitude of the heteronuclear NOE provides a first measurement of backbone HN vector motion, with values larger than 0.65 identifying rigid regions and smaller values corresponding to more flexible regions in the ns-ps timescales. The global analysis of the ^15^N relaxation data was done considering only the 94 amide sites which showed low internal mobility, which were identified as those with a heteronuclear NOE larger than 0.65 and a T_2_ value not significantly reduced relative to the others [30]. This analysis indicates that the homotrimeric ring tumbles as an oblate rotor, with an effective rotational correlation time of ∼40 ns, an anisotropy of 0.11, and with the principal component of the diffusion tensor (of minimal diffusion, for an oblate ellipsoid) closely matching the direction of the highest principal component of the inertial tensor (which coincides with the three-fold symmetry axis of the ring). These results are consistent with values computed by hydrodynamic modeling using HYDRONMR [31].

**Figure 2:**
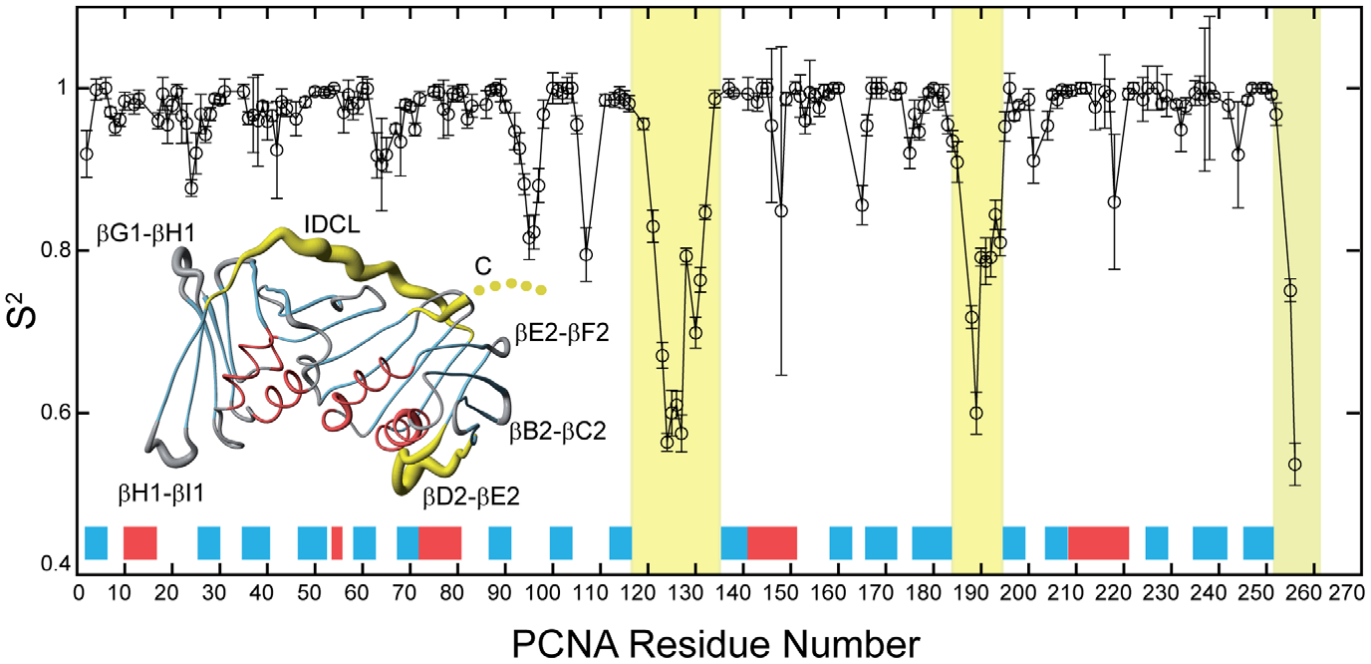
Internal backbone dynamics of PCNA in solution measured by ^15^N relaxation. The order parameter S^2^ for backbone HN bonds are plotted for those residues whose ^15^N T_1_, T_2_, and ^15^N{^1^H} NOE values could be measured and analyzed. The location of the secondary structure elements along the PCNA sequence is indicated by red and blue boxes for α-helices and β-strands, respectively. The last five residues at the C-terminal end are not seen in the crystal structure and therefore an order parameter was not calculated, but these residues have the smallest heteronuclear NOEs and the largest T_2_ times, indicating that they are the most flexible residues in the protein (see Figure S1). The regions of the graph corresponding to the IDCL (residues 117–134), the βD2−βE2 loop (184–195), and the C-terminus (252–261) are shaded in yellow. Inset: Representation of the PCNA backbone structure as a coil whose thickness is proportional to the order parameter S^2^ of the backbone NH bond of the corresponding residue. For simplicity only one of the protomers is shown, but the data correspond to measurements done on the homotrimer. For the residues whose order parameter could not be calculated the thickness was interpolated based on the solid line joining the available values plotted in the graph. Helices and strands are colored in red and blue, and the three most flexible regions are colored in yellow (as in the graph). The loops with high relative disorder are labeled using the same nomenclature as in Figure 1.

The combined analysis of the ^15^N T_1_, T_2_, and ^15^N-{^1^H} NOE values in terms of the model free formalism of Lipari-Szabo [32] allows a more formal description of the HN bond internal dynamics in terms of the order parameter, S^2^. This order parameter provides a measurement of the amplitude of the internal motion in the ns-ps timescales. A value of 1 corresponds to totally immobile, and a value of 0 corresponds to totally unrestricted motion. Figure 2 shows the values of the order parameter for each residue obtained from this analysis. There are several regions in the polypeptide chain of PCNA with increased flexibility with respect to the α-helices and β-strands, which are generally very rigid. These regions are mainly located in the exposed loops connecting secondary structure elements, and particularly in the IDCL and the βD2−βE2 loop, which are the longest loops in the protein. The residues at the C-terminal tail present the highest flexibility (see also Figure S1).

### PIP-box Fragments of p21 Binding to PCNA can be Characterized in Detail by NMR

The CDK inhibitor p21 mediates the cellular response to DNA damage by arresting the cell cycle at the G_1_ phase, inhibiting the progression into the S phase and thus DNA replication [33]. It is an intrinsically disordered protein with an N-terminal sequence similar to other CDK-inhibitory proteins (including p27^Kip1^, p57^Kip2^, and p27^XIC1^) and a C-terminal region containing a PIP-box. The p21 fragments 139–160 (p21^22^) and 141–160 (p21^20^) containing this sequence bind to PCNA with *K_d_* = 83 and 88 nM, respectively, at 30°C [13], [34]. The smallest p21 fragment still competing with p21^20^ for PCNA binding was found to be the 12-mer 141–152 fragment (p21^12^) [34]. The crystal structure of the complex between human PCNA and p21^22^, determined at 2.6 Å resolution, shows electron density only for p21 residues 143–160 [14]. The amino acid sequence of p21^22^ is ^139^GRKRRQTSMTDFYHSKRRLIFS^160^, where the PIP-box canonical residues are underlined.

**Figure 3:**
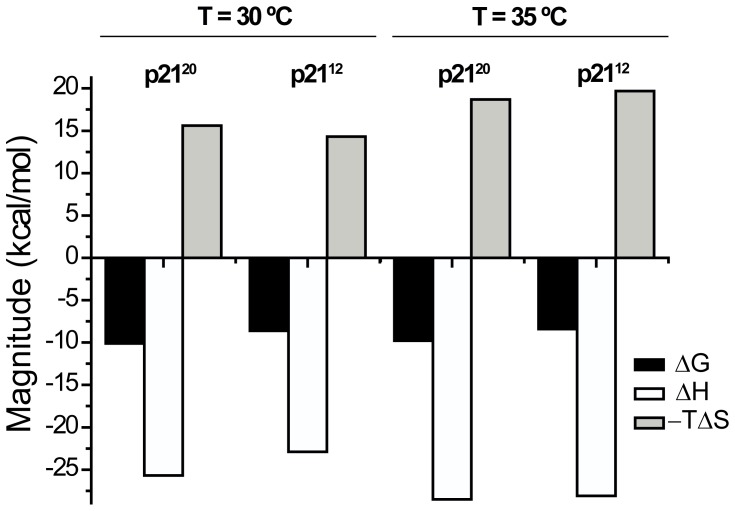
Thermodynamic parameters of the PCNA binding to p21 peptides. The bar diagram shows the change in free energy (ΔG), enthalpy (ΔH), and in the contribution of entropy to the free energy (−TΔS) of the binding of PCNA to the p21^20^ and p21^12^ peptides, as determined by ITC at two temperatures in PBS pH = 7.0. The uncertainty in the experimental values is estimated to be about 5%.

We have characterized the thermodynamics of the p21^20^ and p21^12^ peptides binding to PCNA by Isothermal Titration Calorimetry (ITC) in the same buffer (PBS pH 7.0) and at the same temperature (35°C) as the NMR experiments described below (Figure 3 and Figure S2). To allow comparison with data in the literature for the p21^20^ [34], ITC experiments were also carried out at 30°C. In all cases high quality fits of the data to a one set of sites model were obtained, compatible with the independent binding of one p21 ligand per PCNA protomer, indicating that there is no or very little cooperativity. The dissociation constant obtained for p21^20^ at 30°C (*K_d_* = 54 nM) is similar to that previously reported in PBS (*K_d_* = 88 nM) but there are large differences in the enthalpy and entropy contributions to the free energy of binding [34]. We do not know the reason for this discrepancy. Our thermodynamic measurements are however very similar to those of the p21^22^ peptide also at 30°C [13]. At 35°C p21^20^ binds PCNA with a *K_d_* = 100 nM, and shortening of the peptide leads to a reduction in affinity at both 30°C (*K_d_* = 640 nM) and 35°C (*K_d_* = 1100 nM).

The NMR ^1^H-^15^N correlation spectrum of human PCNA in the presence of the p21^20^ peptide shows many signals experiencing CSPs (Figure S3). However, the low solubility of this complex limited the sensitivity of triple resonance experiments and the spectrum could not be assigned by this means. Therefore we first carried out a detailed study of the spectrum of the complex with the shorter p21^12^ peptide (Figure S4), which is more soluble. As described in the methods sections, a titration of PCNA with a highly concentrated stock of the p21^12^ peptide made it possible to follow the shift of most of the PCNA signals upon peptide binding (Figure S5), and a series of triple resonance experiments confirmed the sequence specific assignment of PCNA bound to p21^12^. This information was then used to assign the ^1^H-^15^N correlation spectrum of PCNA bound to the longer p21^20^ peptide.

The secondary structure of PCNA, determined by the chemical shifts analysis using TALOS+ [35], does not change when bound to the p21^12^ peptide (data not shown). Just for a few isolated residues the assignment as helix, extended or loop is changed, and this occurs almost always at the edges of helices or strands. There is only one instance where two consecutive residues change their assignment from loop to extended. This occurs for residues 255 and 256, at the C-terminal end of PCNA (which is 261 residues long but only up to residue 256 is visible in the crystal structure of the complex). These two residues experience large CSPs upon peptide binding (see below). The secondary structure assignment of the IDCL residues of PCNA (amino acids 117–135) is EEEEEEELEEELLLLLLL (with E meaning extended and L meaning loop), and it is identical in free and bound PCNA. This result is consistent with the crystal structures shown in Figure 1. Although this figure appears to indicate otherwise, there is no major change in the structure of the IDCL upon binding to p21. In fact, in all available crystal structures of PCNA the IDCL sequence has an extended conformation, but only in complexes with PIP ligands are the residues in the N-terminal half (approximately) of the IDCL involved in intermolecular hydrogen bonds with the pattern corresponding to a canonical β-sheet structure. For this reason the secondary structure of the IDCL is classified as coil in free PCNA and as a β-strand in the complex by the Define Secondary Structure of Proteins program (DSSP) used by the Protein Data Bank [36]. There are some local changes at the IDCL upon p21 binding, but they are small. The average root mean square deviation for the C_α_ atoms of PCNA residues 117–134 in the bound form (PDB file 1ACX) is 0.61 Å when superimposed on the corresponding residues of free PCNA in the PDB file 1W60. The RMSD for the same atoms and residues is 1.63 Å when the superimposition is done using the free PCNA structure 1VYM.

**Figure 4:**
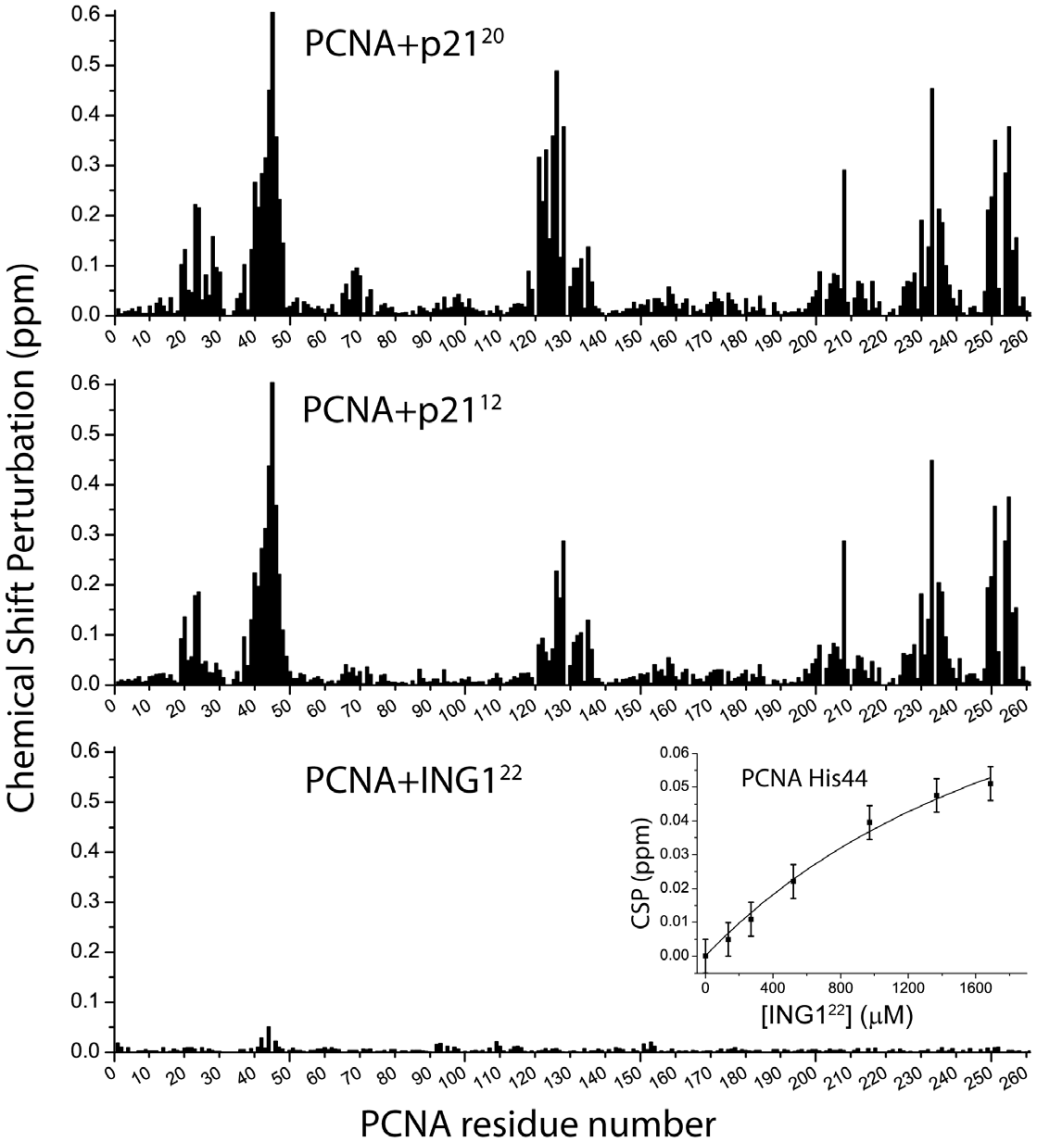
Chemical shift perturbations of PCNA backbone amide resonances caused by PIP-box peptides. The top and middle panels show the CSP values for each PCNA residue in the presence of a 1.2 or 1.6 excess (on a monomer basis) of p21^20^ or p21^12^ peptides, respectively. Under the experimental conditions used PCNA is saturated with the peptides (we calculate that 99.9 and 99.8% of the PCNA molecules are bound to the p21^20^ or the p21^12^ peptide, respectively). The bottom panel represents the CSP values in the presence of a 7.8 excess of the ING1^22^ peptide. The inset shows the CSP experienced by PCNA residue H44 at increasing concentrations of the peptide with error bars spanning twice the estimated uncertainty in the CSP values (±0.005 ppm), which is the average CSP value. The line is the best fit to a model of one set of identical binding sites.

The CSPs are represented for each residue in Figure 4, and the residues experiencing the largest perturbations are indicated in the structure of the complex with the p21^22^ peptide in Figure 1B. The CSPs delineate a clear region of peptide binding that is in very good agreement with the crystal structure. The pattern of the CSPs measured in PCNA bound to the p21^12^ peptide is very similar to that measured when bound to p21^20^ (Figure 4 and S6). The main difference is the smaller magnitude of the values for residues 121–128, which is the part of the IDCL in close contact with the βB strand of p21^22^ (as seen in the crystal structure), mostly absent in p21^12^ (Figure 1B). Smaller differences are seen in residues located not far away from this part of the IDCL, like residues 23–30, 65–73 and 118–120, as can be seen in the difference CSP plot in Figure S6.

The heteronuclear ^15^N-{^1^H} NOEs of PCNA saturated with the p21^12^ peptide (Figure S1) show that most of the PCNA regions with high relative flexibility in free PCNA remain so in the complex, including the portion of the IDCL that directly interacts with this peptide. The exception is the C-terminal region, which experiences a reduction in its flexibility. The low solubility of PCNA bound to p21^20^ prevented a reliable measurement of the heteronuclear NOEs in this complex.

### The ING1 PIP-box Fragment Binds Very Weakly to PCNA

ING1 is a member of the INhibitor of Growth family of tumor suppressors that induces apoptosis in response to DNA damage [37] and contributes to the epigenetic control of cellular senescence [38]. ING1 is expressed in human cells in four isoforms, the major one of them (named p33ING1b) containing a PIP-box sequence in its N-terminal region [39]. It has been reported that ING1 binds PCNA (as observed by co-immunoprecipitation), and that binding is increased in response to UV irradiation [16]. This binding is specifically inhibited by overexpression of p21, but not by the CDK2 inhibitor p16^MTS1^, which has no PIP sequence. ING1 PIP deletion mutants do not bind PCNA and do not induce apoptosis, suggesting a role in eliminating UV-damaged cells through programmed cell death.

In order to characterize the binding of ING1 PIP-box we have used the fragment ^4^PANGEQLHLVNYVEDTLDSIES^25^ (ING1^22^, with PIP-box residues underlined). A titration of PCNA with this peptide up to a 7.8-fold molar excess produced CSPs that were much smaller than those measured for the p21 peptides (Figure 4). A cluster of PCNA residues in the region 42–45 show CSP values above the average with H44 showing the largest perturbation. The CSPs measured for the PCNA H44 residue along the titration (see inset in Figure 4) can be fitted to a model of one set of sites with a dissociation constant *K_d_* = 2.0±0.7 mM. If the fitting is made with the CSP value at infinite peptide concentration (CSP_max_) as a constant equal to the CSP value measured in the spectra of PCNA bound to p21^20^, then *K_d_* = 12.5±0.5 mM. Therefore the binding of ING1^22^ to the PIP site of PCNA is extremely weak, with an affinity about 4–5 orders of magnitude smaller than that of p21^20^.

### Direct Binding of MCL-1 Protein to PCNA is not Detected in Solution

MCL-1 is a member of the pro-survival B-cell lymphoma-leukemia-2 (Bcl-2) family that preserves mitochondrial integrity during apoptosis [40]. It consists of an intrinsically disordered, 170 residue long N-terminal region and a C-terminal globular domain with three Bcl-2 homology (BH) regions [41]. MCL-1 was reported to interact with PCNA through a PIP-box (^221^ QRNHETAF ^228^), located at the end of the BH3 region [18]. This interaction was observed by co-immunoprecipitation, and was found to inhibit cell-cycle progression through the S-phase, revealing a dual role of MCL-1 as a pro-survival protein and as an inhibitor of the cell cycle.

We have monitored the binding of MCL-1 to PCNA by NMR using an MCL-1 construct that lacks most of the disordered N-terminal region but retains the C-terminal domain correctly folded [19]. This fragment contains the MCL-1 residues 152–343 plus an N-terminal histidine-tag, and was named ΔN151−ΔC7 in the original publication [19]. In the TROSY spectra of PCNA in the presence of 4.4 equivalents of this MCL-1 construct (Figure S7) we did not observe any indication of binding based on backbone amide chemical shift or intensity changes. As no significant CSPs were measured, it is not possible to confirm the interaction, but a lower limit for the dissociation constant of the putative complex can be estimated. Assuming an equimolar complex, and that we should be able to detect significant changes when at least 20% of PCNA is bound to MCL-1 [42], a lower limit of the dissociation constant can be calculated [43]. Under the experimental conditions used, the dissociation constant for the possible PCNA/MCL-1 complex is larger than 0.84 mM.

### Direct Binding of CDK2 Protein to PCNA is not Detected in Solution

PCNA has been detected in immunoprecipitates containing p21, several CDKs, and their regulatory cyclins [44]. The structure of the CDK2/cyclin-A complex with the p21 fragment ^155^RRLIF^159^ shows that this p21 peptide binds to the cyclin groove and it does not interact with CDK2 [45]. The similarity of the structure of this p21 sequence bound to the cyclin and bound to PCNA (as part of the p21^22^ 139–160 fragment) suggests that p21 acts as a double-sided sticky tape, with one face contacting PCNA and the other contacting cyclin-A [46]. This model implies that there is no direct interaction between PCNA and CDK2, which is not consistent with the direct interaction observed in pull down and surface plasmon resonance (SPR) experiments using recombinantly produced GST-CDK2 and PCNA proteins [17]. The observation that a GST fusion of a C-terminal p21 fragment, which by itself does not interact with CDK2, is able to pull-down CDK2 together with PCNA from cell lysates is consistent with CDK2 interacting with PCNA [47]. However, in this experiment the simultaneous presence of cyclin-A was not investigated.

To confirm if CDK2 and PCNA proteins interact directly when mixed in solution we have tried to observe the binding by NMR. In the TROSY spectra of PCNA in the presence of 1 equivalent of CDK2 we did not observe any indication of binding based on backbone amide backbone amide chemical shift or intensity changes (Figure S8). This suggests that the direct interaction, if any, occurs with very low affinity. Under the experimental conditions used, we estimate that *K_d_*>0.16 mM.

## Discussion

Some of the loops of PCNA are highly dynamic in the ns to ps time scales, in particular the IDCL and the βD2−βE2 loop. The last 8 residues in the C-terminal tail present the highest flexibility, consistent with most of them not appearing in the electron density map of the crystallographic structures. The relative high flexibility observed in the loops is in general agreement with the high crystallographic B-factors for residues in the corresponding regions of the X-ray structures of free PCNA [46]. However, there is a large variability in the B-factors reported for two different crystal forms. In the monoclinic form (PDB entry 1VYM) both the IDCL and the βD2−βE2 loops display very large B-factors as compared with the average, while in the trigonal form (PDB entry 1W60) only the βD2−βE2 loop shows relative high values. This discrepancy is very likely due to the different number of crystal contacts involving the IDCL residues, as evaluated by the program CRYCO [48]. There are more than 50 contacts in each of the PCNA chains in 1W60 and only between 0 and 28 contacts in the chains in 1VYM.

In the complex with the p21^22^ fragment (PDB 1AXC) the βD2−βE2 loop still stands out with large B-factors (and four residues in the middle are not even seen) while the IDCL does not. However the assumed rigidification of the IDCL upon peptide binding is unclear since there are around 50 crystal contacts per PCNA chain involving the IDCL residues in this crystal. A loss of flexibility at the IDCL is observed in the crystal structure of PCNA bound to a 16-residue long peptide designed with a consensus sequence and named PL [46]. In this structure (PDB entry 1VYJ) the IDCL residues are involved in few or no crystal contacts (between 28 and 0, depending of the chain). Therefore, there is a rigidification of the IDCL upon binding to PL that we do not observe in solution when the IDCL is bound to p21^12^. The reason for this discrepancy might be that p21^12^ is three-residue shorter at its C-terminus than PL, or that there is little change in the dynamics at the time scale that can be observed by the ^15^N-{^1^H} NOE (ps-ns), or both. We do observe in solution that upon p21^12^ binding there is a reduction in the dynamics of the PCNA C-terminal residues.

We have been able to observe the p21^20^ peptide binding to the PCNA ring in solution by chemical shift perturbation analysis, including a delineation of the binding site. The binding is consistent with the crystal structure of the complex with the p21^22^ peptide but subtle differences are observed. The last six residues of PCNA were not observed in the crystal structure of the complex [14], and were proposed to be involved in loose electrostatic contacts with the (also not observed) first four residues of the p21^22^ peptide. Interestingly, two of those six PCNA residues experience large CSPs in solution suggesting that they are involved in interactions with p21 that are more persistent than the others (see Figure 1B). Most of the PCNA residues experiencing the largest CSPs are at short distances from the p21 peptide (as seen in the crystal structure), but large CSPs are also observed in some distant PCNA regions, for example the PCNA 19–24 residues, a loop with intermolecular Cα-Cα distances larger than 15 Å. Furthermore, there are many PCNA residues with small but significant CSPs (above the average CSP measured with the ING1^22^ peptide). These long range effects may be due to small conformational changes that affect the extremely sensitive amide chemical shifts. However these changes are not detected by the comparison of the PCNA free and bound crystal structures, which superpose with a root mean square deviation for the Cα atoms of 0.61 Å. It is possible that in solution there are a number of very similar PCNA structural states in equilibrium whose relative populations change upon peptide binding, while only one is captured in the crystal lattice. Interestingly, we have previously shown that human PCNA is highly dynamic in the s to ms time scales as evaluated by amide hydrogen solvent exchange, in contrast to yeast PCNA despite having essentially identical structures [49].

The pattern of the CSPs caused by p21^12^ binding is very similar to those caused by p21^20^ (Figure 4 and S6), which demonstrates that the structure of the two complexes is basically the same. The main difference is the smaller magnitude of the CSPs for residues 121–128, the part of the IDCL that interacts with the βB strand of p21, which is mostly absent in the p21^12^ peptide (Figure 1B). Our results show that this shorter peptide retains most of the molecular recognition determinants of the PCNA interaction with p21, and explains why this was the shortest peptide able to compete with p21^20^ for binding to PCNA [34]. Still the affinity is reduced by one order of magnitude, as we have measured by ITC. The analysis of the enthalpic and entropic components of the binding Gibbs energy indicates that the balance of forces determining this reduced binding affinity varies with temperature. At 30°C, shortening of the peptide affects both the enthalpic and entropic contributions, although the reduction of the unfavorable binding entropy does not compensate the loss of favorable enthalpic contributions. At 35°C the effect is mostly of entropic nature with the binding enthalpy of both peptides being very similar.

In contrast with p21, the PIP-box peptide of the tumor suppressor ING1 binds PCNA with a very low affinity, about 4–5 orders of magnitude smaller affinity than the p21^20^ peptide. Because of the extremely high sensitivity of the NMR chemical shifts we could detect the binding through the observation of changes in the resonances of PCNA residues in the region 42–45, which experience the largest CSPs when bound to p21 peptides. These residues form a solvent exposed loop in PCNA and are very close to the p21 αA helix in the complex. The observation that these same residues experience the largest CSPs in the presence of the ING1^22^ peptide demonstrate that the binding site is the same as the p21, at least for the PIP-box sequence forming the αA helix. However, the extremely low affinity suggests that the binding observed in cell lysates by co-immunoprecipitation is mediated by other factors. The reason for such a low affinity for the ING1^22^ peptide may be that its PIP-box sequence (^9^ QLHLVNYV^16^) lacks the second aromatic residue.

The scientific literature reports a wide variety of PIP motifs in proteins that interact with PCNA, and a less stringent consensus has been suggested [18] without restrictions at the fourth and seventh positions (*QXXXXXXa*, where *a* is F or Y, and *X* is any of the 20 proteinogenic amino acids). MCL-1 has a sequence that meets this minimal PIP-box motif (^221^ QRNHETAF ^228^) at the end of the BH3 region in its globular C-terminal domain. Its interaction with PCNA was detected by co-immunoprecipitation in lysates from co-transfected human embryonic cells and also from native HeLa cells, and was confirmed by mutational analysis of the PIP-box minimal sequence [18]. However we did not detect binding of PCNA to the Bcl-2 domain of MCL-1 in solution, suggesting that the interaction, if any, occurs with very low affinity or is mediated by other factors. The full length MCL-1 protein consists of an intrinsically disordered, 170 residue long, N-terminal sequence with two regions enriched in proline, glutamate, serine and threonine residues (PEST regions), which are responsible for the rapid turnover of the protein and for its cellular localization [50]. Truncation at the N-terminus generates an isoform with higher stability and abundant in tumors [51]. It is possible that the N-terminal region (absent in the MCL-1 construct used here), and/or that another unidentified biomolecule, cooperate in the binding of MCL-1 to PCNA observed in cell lysates. Still it is surprising that a yeast two-hybrid assay indicated that the C-terminal fragment 137–261 of PCNA (residues 137–261) was sufficient for the interaction with MCL-1 [18] since this truncated PCNA chain is unlikely to form a well folded protein and the functional homotrimeric rings.

Our observation of no or weak binding of the ING1^22^ peptide and the MCL-1 C-terminal domain to PCNA is not consistent with the proposed less stringent consensus PIP-box sequence. On the contrary, a more stringent one is necessary to explain the affinity of the different PIP-box sequences, as discussed below.

The high affinity between p21 and PCNA is unusual among proteins containing the PIP-box motif. The p21^22^ fragment binds PCNA with a much higher affinity than the p66^22^ or Fen-1^22^ fragments (*K_d_* = 0.08, 1.5 and 60 µM, respectively) probably because of a more efficient hydrophobic packing of the αA helix and more favorable electrostatic interactions with the C-terminal region of PCNA [13]. A competition analysis of variants of the p21 PIP-box sequence showed that the ^144^ QTSMTDFY ^151^ peptide alone did not compete with p21^20^ for PCNA binding, while the ^141^KRRQTSMTDFYH^152^ (p21^12^) did, suggesting that the basic residues flanking the N- and C-termini contribute to the binding [34]. The sequences flanking the PIP-boxes of p66 and Fen1 peptides have progressively less basic residues and their affinities decrease accordingly [13]. A consensus sequence with an N-terminal flanking region without basic residues binds PCNA with an affinity similar to the p21^20^ peptide, suggesting that the C-terminal ones contribute the most to the affinity.

The structure of PCNA bound to the Fen-1 protein shows that the C-terminus of Fen-1 is engaged in more contacts with PCNA than the Fen-1^20^ peptide is in the corresponding crystal structure, and there are additional contacts involving the core domain of Fen-1. These differences explain the approximately three orders of magnitude higher affinity measured for the full length protein (see below). The affinity for the binding of the full-length p21 protein has not been measured, but since it is an intrinsically disordered protein [52], and assuming that it contains no other binding sites besides the PIP-box sequence, its affinity might be close to that of the longest C-terminal fragment tested. This fragment contains residues 87–164, and binds PCNA with a *K_d_* = 15 nM at 37°C [53]. This number is likely underestimated because the p21 fragment was fused to GST, which forms homodimers and may cause an apparent increase in affinity in pull-down binding experiments [54]. However it is consistent with the observed displacement of the Fen-1 protein from PCNA rings by p21 fragments, since Fen-1 binds with *K_d_* = 60 nM, as measured by SPR at 25°C [55]. Furthermore, binding of the p21 fragment and the Fen-1 protein to the PCNA ring was found to be mutually exclusive. This observation suggested that binding induced a conformational change such that the trimer could bind either p21 or Fen-1 at all three sites. However, the structures of PCNA bound to different proteins and peptides determined afterwards showed only small changes in some loops and the C-terminus. It is possible that the high affinity of p21 for human PCNA is uniquely high and necessary for its blocking of the DNA replication after cellular stress. Still the binding affinity of some PIP-box containing proteins for PCNA may depend more on the interaction with the DNA (direct or mediated by third party factors) than on the presence of additional protein-protein contact points outside the PIP box. Interestingly it has been shown that the sequence Thr-Asp between the hydrophobic residue and the two aromatic residues of the canonical PIP-box sequence (called the TD motif) increases the binding of PIP-box containing proteins to chromatin-bound PCNA [56]. This motif is present in p21 and partially in Fen-1 (Asp-Asp sequence), and p66 (Thr-Gly). The TD residues are exposed to the solvent and do not establish interactions with PCNA in the crystal structures of the complexes, but the situation may be different in the context of the chromatin, where transient interactions with the DNA and/or chromatin associated proteins may occur [57]. The TD motif is not present in the ING1 or MCL-1 PIB-box sequences.

We have been unable to detect any binding of CDK2 to PCNA in solution by NMR, indicating that if they interact directly they do it very weakly. This result is inconsistent with the report of a direct PCNA/CDK2 interaction by pull-downs and SPR measurements using recombinantly produced GST-CKD2 and PCNA proteins [17], but is consistent with the model derived from the structures of the CDK2/cyclin-A/p21-^155^RRLIF^159^, and PCNA/p21^22^ complexes, which excludes a direct PCNA-CDK2 interaction [46]. Both CDK2 and PCNA have been crystallized alone or bound to different ligands, but there is no report on the structure of a binary CDK2/PCNA complex. CDK2 does not contain a PIP-box motif. Interestingly, the direct interaction between the Growth arrest and DNA damage 45 alpha protein (Gadd45α), also with no PIP-box motif, was recently found to be extremely weak in solution [58].

PCNA inhibitors are currently being developed a potential anticancer agents, and recently the crystal structure of PCNA with a small molecule inhibitor bound to the PIP-box binding site has been reported [59]. Our work shows that NMR can now be used to study ligand binding to the 90 kDa PCNA ring, opening a new way to investigate direct interactions in solution.

## Supporting Information

Figure S1 **Changes in PCNA backbone dynamics upon p21^12^ binding.** Backbone amide ^15^N NMR relaxation parameters for PCNA at 60 MHz in PBS pH 7.0 at 35°C. The heteronuclear {^1^H}-^15^N NOEs, and ^15^N transversal (*T_2_*) and longitudinal (*T_1_*) relaxation times are represented for each residue of PCNA in its free form (black open circles) and, in the case of the {^1^H}-^15^N NOEs, also bound to p21^12^ peptide (red open circles).
(TIF)

Figure S2 **Calorimetric titrations of PCNA with p21 peptides at 30 and 35**°C**.** For each graph the upper panels represent the heat effect associated with the peptide injections and the lower panels represent the ligand concentration dependence of the heat released upon binding, after normalization and correction for the heats of dilution. In the lower panels the symbols are the experimental data, and the continuous line is the best fit to a model of one set of identical binding sites.
(TIF)

Figure S3 **PCNA binding to p21^20^ peptide in solution observed by NMR.** Overlay of the ^1^H-^15^N TROSY spectra of free PCNA (black) and PCNA bound to the p21^20^ (red) peptide. These two spectra were measured at 800 MHz and 35°C on a 125 µM PCNA sample in PBS pH 7.4, with a 1∶3 PCNA:p21 peptide ratio, on a monomer basis.
(TIF)

Figure S4 **PCNA binding to p21^12^ peptide in solution observed by NMR.** Overlay of the ^1^H-^15^N TROSY spectra of free PCNA (black) and PCNA bound to the p21^12^ (red) peptide. These two spectra were measured at 800 MHZ and 35°C on a 125 µM PCNA sample in PBS pH 7.4, with a 1∶11 PCNA:p21 peptide ratio, on a monomer basis.
(TIF)

Figure S5 **Titration of PCNA with the p21^12^ peptide.** The two panels show representative regions of thirteen superimposed ^1^H-^15^N HSQC spectra of 0.9 mM of uniformly labeled ^2^H,^13^C,^15^N-PCNA in the presence of increasing concentrations of p21^12^ peptide (the different colors correspond to peptide concentration values from 0 to 1.3 mM in 0.1 mM increments). The arrows indicate the signal movement during the titration for selected amide resonances: residues 12, 13, 78, 203 and 214 (top panel) are in fast exchange, residue 158 (bottom panel) in intermediate exchange, and residues 230 and 250 (right panel) in slow exchange.
(TIF)

Figure S6 **Differences in the CSP of PCNA bound to p21^20^ or to p21^12^ peptide.** These CSP values were calculated from the chemical shifts measured on spectra of PCNA bound to p21^20^ and using as reference the chemical shifts of PCNA bound to p21^12^.
(TIF)

Figure S7 **NMR examination of the interaction of PCNA with MCL-1.** ^1^H-^15^N TROSY spectra of 50 µM uniformly labeled ^2^H-^15^N PCNA alone (black) or in the presence (red) of 220 µM of the ΔN151−ΔC7 fragment of human MCL-1 (1∶4.4 ratio on a PCNA monomer basis). The spectra were recorded in 10 mM Tris pH 7.4, 250 mM NaCl, 1 mM EDTA, 0.5 mM DTT at 800 MHz and 35°C. The weak red signals around 8.3 and 122 ppm in the ^1^H and ^15^N chemical shift dimensions, respectively, arise from degradation products of PCNA. This interpretation is based on the observation that their chemical shifts are typical of random coil polypeptides, and that their number and intensity increase over time. This interpretation was confirmed by SDS-PAGE and mass spectrometry analysis of the NMR sample. We think that PCNA proteolysis is caused by traces of proteases that co-purified with the MCL-1 protein. This experiment was repeated using a different batch of purified MCL-1 protein with the same results. A small systematic reduction in the intensity of the PCNA signals was observed in the spectrum of the mixture relative to PCNA alone, which can be explained by contributions from i) the decrease in the concentration of intact PCNA protein over time due to proteolysis, ii) the increase in the medium viscosity causing a slower tumbling of the protein, and iii) a possible non-specific binding into large aggregates that are not visible by NMR. Similar observations were made on samples with different molar ratios of the two proteins and a with a shorter MCL-1 fragment (named the core domain of MCL-1, cMCL-1 or ΔN162−ΔC24) which contains residues 162–327 (data not shown). If MCL-1 were specifically bound to PCNA, a localized, non-uniform signal intensity reduction, in addition to chemical shift perturbations, should be observed in the PCNA signals.
(TIF)

Figure S8 **NMR examination of the interaction of PCNA with CDK2.** ^1^H-^15^N TROSY spectra of uniformly labeled 50 µM ^2^H-^15^N PCNA alone (black) or in the presence (red) of CDK2 (1∶1 ratio on a PCNA monomer basis). The spectra were recorded in 20 mM Tris pH 7.2, 150 mM NaCl, 1 mM DTT at 800 MHz and 35°C.
(TIF)
