# Correction: Proliferating Cell Nuclear Antigen (PCNA) Interactions in Solution Studied by NMR

**DOI:** 10.1371/journal.pone.0095818

**Published:** 2014-04-18

**Authors:** The PLOS ONE Staff

The legend of Figure 2 is incorrect; the thickness of the PCNA backbone structure as a coil is not proportional to S^2^ but is related to 1-S^2^, using an arbitrary scaling factor and an offset that produced a visually informative figure using the graphics program MolMol. The exact relationship is: MolMol_thickness = ((1-S^2^)*3)+0.2. Please see the corrected Figure 2 legend here.

**Figure 2:**
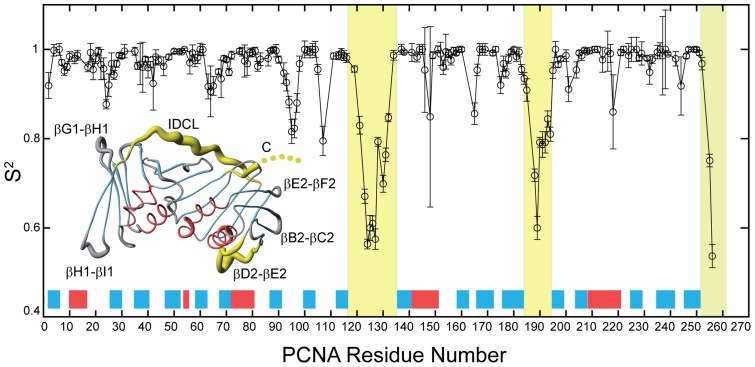
Internal backbone dynamics of PCNA in solution measured by ^15^N relaxation. The order parameter S^2^ for backbone HN bonds are plotted for those residues whose ^15^N T_1_, T_2_, and ^15^N{^1^H} NOE values could be measured and analyzed. The location of the secondary structure elements along the PCNA sequence is indicated by red and blue boxes for α-helices and β-strands, respectively. The last five residues at the C-terminal end are not seen in the crystal structure and therefore an order parameter was not calculated, but these residues have the smallest heteronuclear NOEs and the largest T_2_ times, indicating that they are the most flexible residues in the protein (see Figure S1). The regions of the graph corresponding to the IDCL (residues 117–134), the βD2−βE2 loop (184–195), and the C-terminus (252–261) are shaded in yellow. Inset: Representation of the PCNA backbone structure as a coil whose thickness is proportional to (1-S^2^)*3+0.2, where S^2^ is the order parameter of the backbone NH bond of the corresponding residue. The values of the scaling factor and offset were arbitrarily chosen to produce a visually informative figure using the program MolMol. For simplicity only one of the protomers is shown, but the data correspond to measurements done on the homotrimer. For the residues whose order parameter could not be calculated the thickness was interpolated based on the solid line joining the available values plotted in the graph. Helices and strands are colored in red and blue, and the three most flexible regions are colored in yellow (as in the graph). The loops with high relative disorder are labeled using the same nomenclature as in Figure 1.

In the upper panel of Figure S1, the ^15^N-{^1^H} NOEs for PCNA bound to the p21 peptide are missing. The authors have provided a corrected version here.

In each of the four panels of Figure S2, the association constant (K) numerical values are correct, but the units (M) are not. The correct units are M^-1^ (molar to the power of minus 1). The authors have provided a corrected version here.

## Supporting Information

Figure S1 **Changes in PCNA backbone dynamics upon p21^12^ binding.** Backbone amide ^15^N NMR relaxation parameters for PCNA at 60 MHz in PBS pH 7.0 at 35°C. The heteronuclear {^1^H}-^15^N NOEs, and ^15^N transversal (*T_2_*) and longitudinal (*T_1_*) relaxation times are represented for each residue of PCNA in its free form (black open circles) and, in the case of the {^1^H}-^15^N NOEs, also bound to p21^12^ peptide (red open circles).
(TIF)

Figure S2 **Calorimetric titrations of PCNA with p21 peptides at 30 and 35°C.** For each graph the upper panels represent the heat effect associated with the peptide injections and the lower panels represent the ligand concentration dependence of the heat released upon binding, after normalization and correction for the heats of dilution. In the lower panels the symbols are the experimental data, and the continuous line is the best fit to a model of one set of identical binding sites.
(TIF)
