# Supplementary figures and images for: Proliferating Cell Nuclear Antigen (PCNA) Interactions in Solution Studied by NMR
Source: PLoS One. 2012 Nov 6;7(11):e48390. doi: 10.1371/journal.pone.0048390 (PMC3491057; doi:10.1371/journal.pone.0048390)

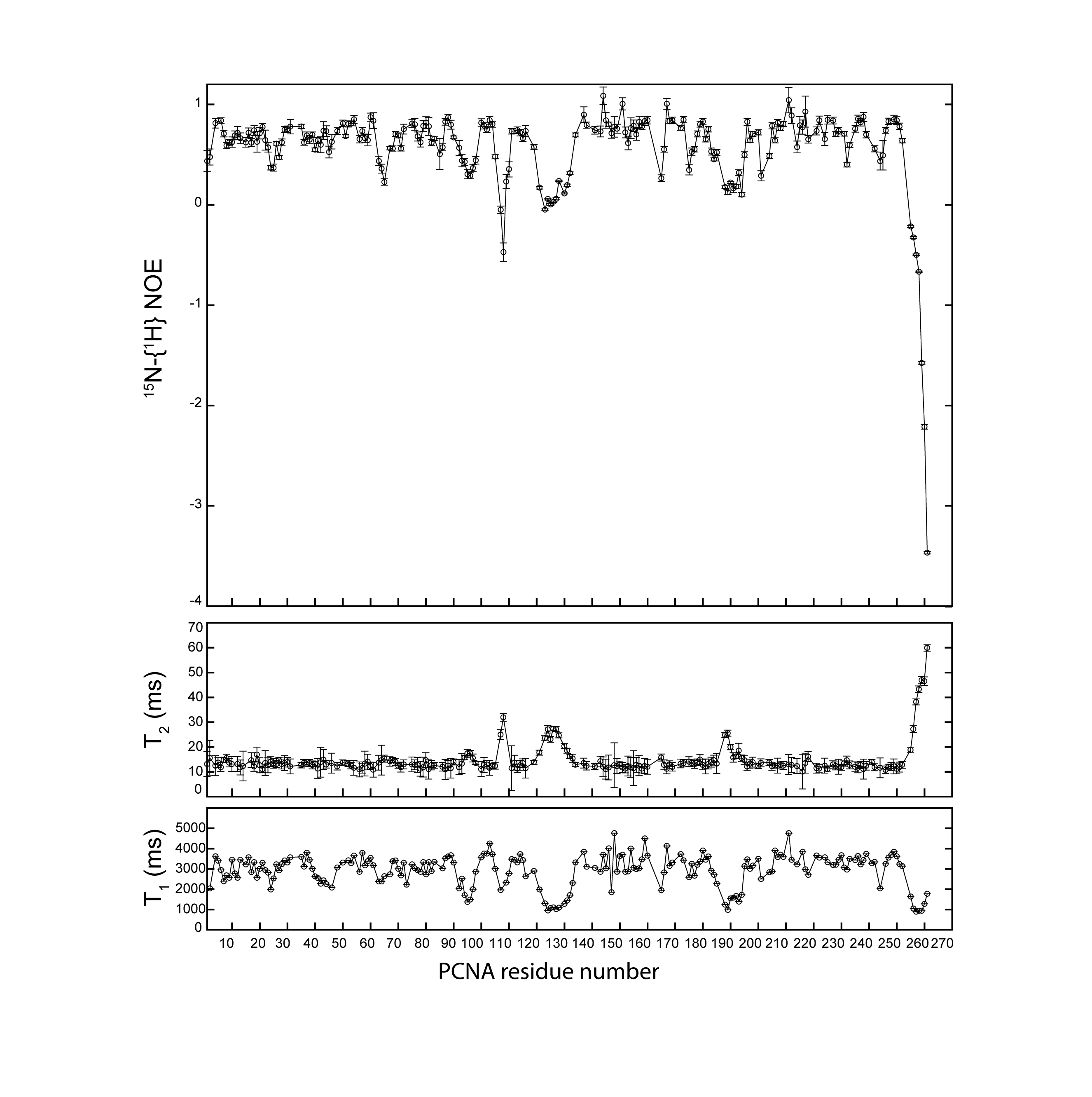

Supplement: Figure S1 — Changes in PCNA backbone dynamics upon p2112 binding. Backbone amide 15N NMR relaxation parameters for PCNA at 60 MHz in PBS pH 7.0 at 35°C. The heteronuclear {1H}-15N NOEs, and 15N transversal (T2) and longitudinal (T1) relaxation times are represented for each residue of PCNA in its free form (black open circles) and, in the case of the {1H}-15N NOEs, also bound to p2112 peptide (red open circles). (TIF) [file pone.0048390.s001.tif]

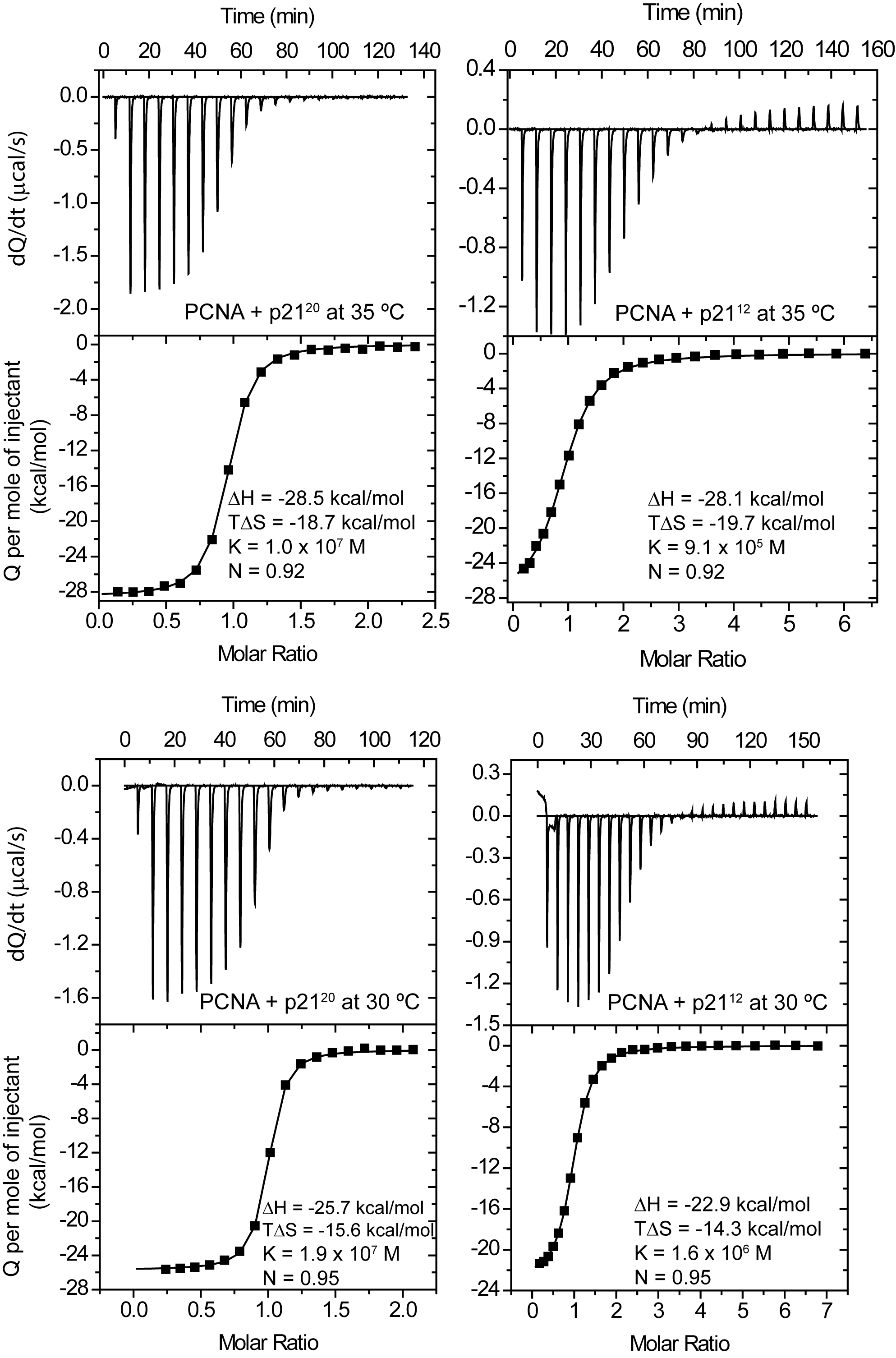

Supplement: Figure S2 — Calorimetric titrations of PCNA with p21 peptides at 30 and 35°C. For each graph the upper panels represent the heat effect associated with the peptide injections and the lower panels represent the ligand concentration dependence of the heat released upon binding, after normalization and correction for the heats of dilution. In the lower panels the symbols are the experimental data, and the continuous line is the best fit to a model of one set of identical binding sites. (TIF) [file pone.0048390.s002.tif]

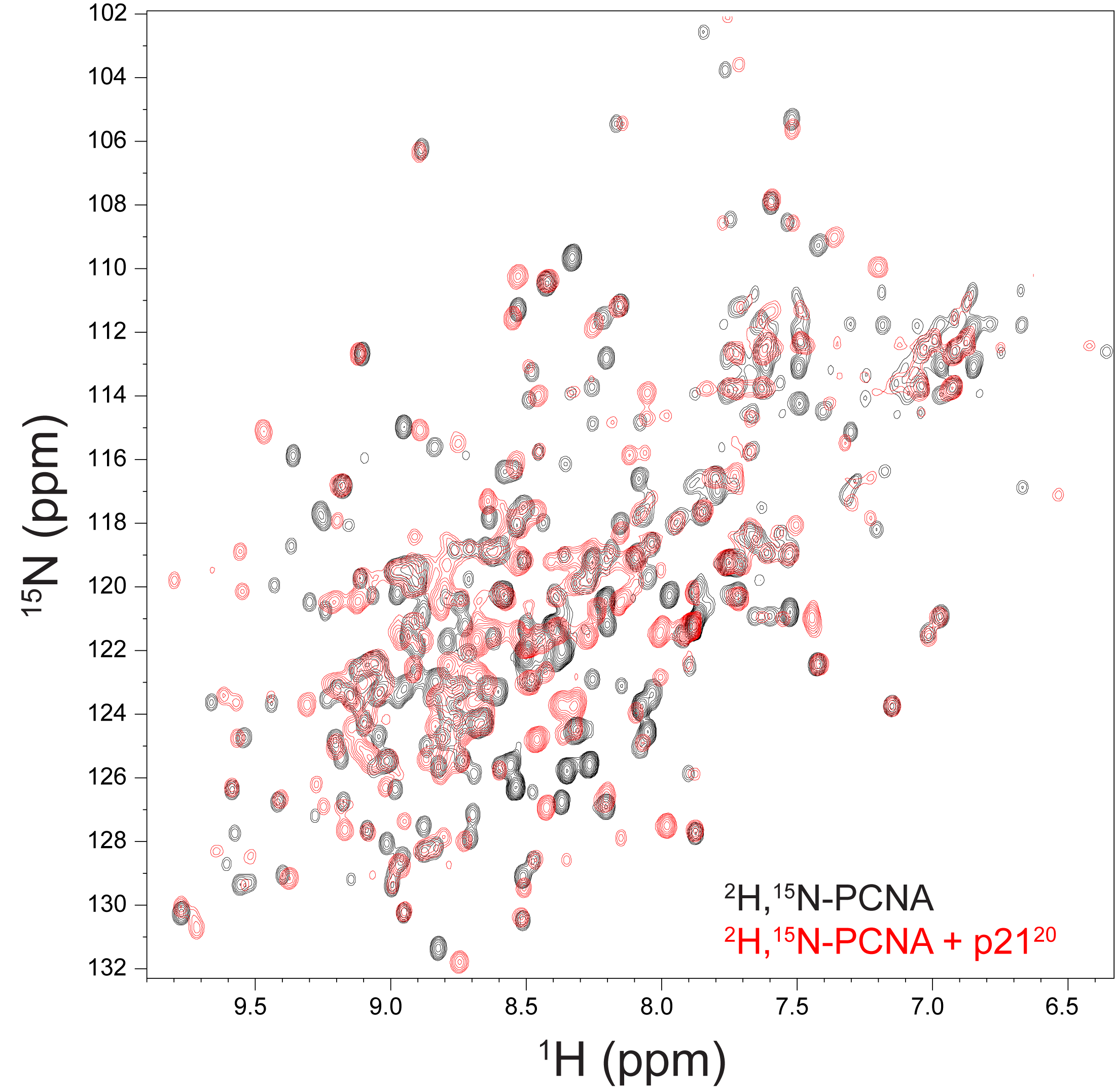

Supplement: Figure S3 — PCNA binding to p2120 peptide in solution observed by NMR. Overlay of the 1H-15N TROSY spectra of free PCNA (black) and PCNA bound to the p2120 (red) peptide. These two spectra were measured at 800 MHz and 35°C on a 125 µM PCNA sample in PBS pH 7.4, with a 1∶3 PCNA:p21 peptide ratio, on a monomer basis. (TIF) [file pone.0048390.s003.tif]

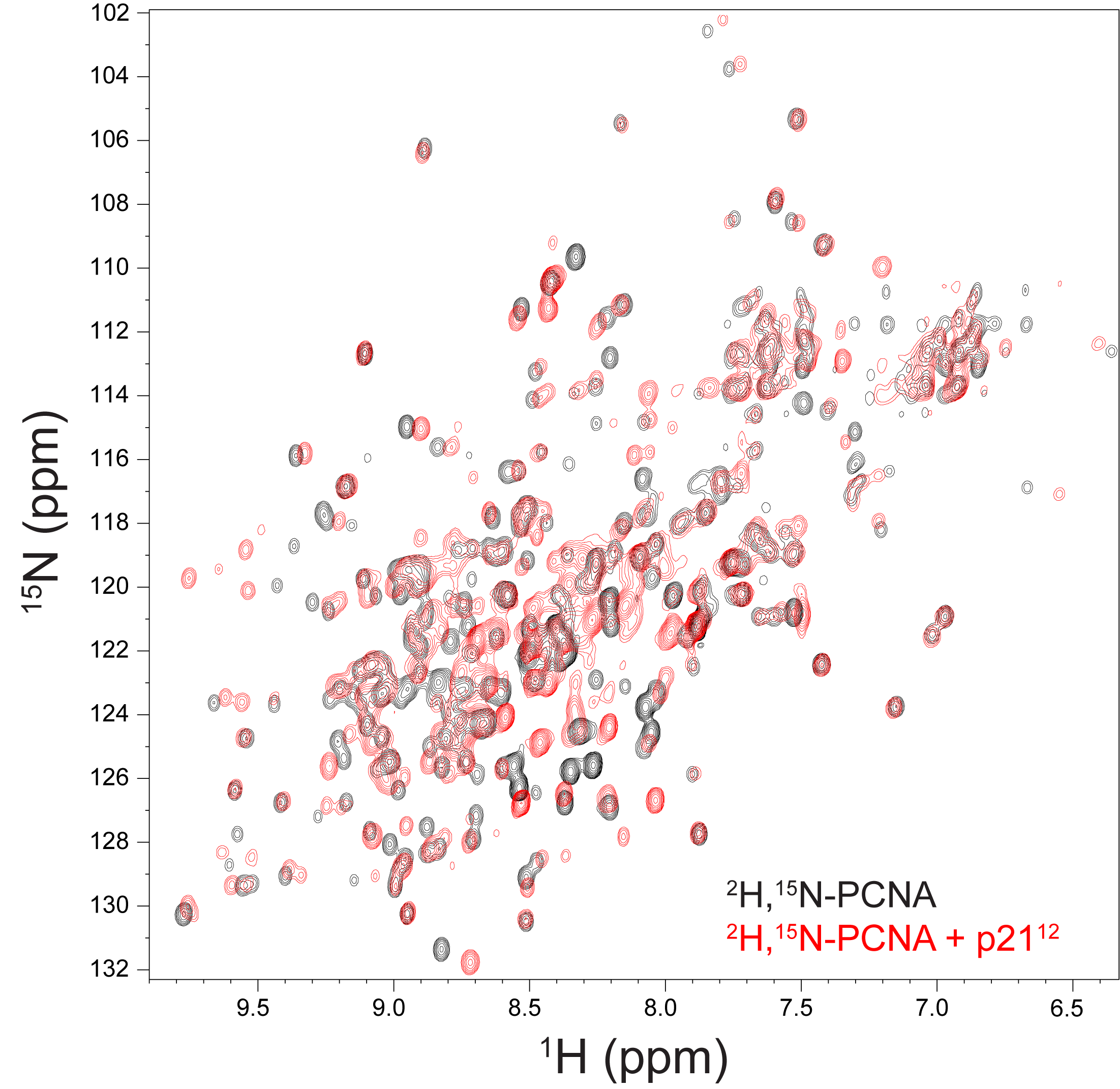

Supplement: Figure S4 — PCNA binding to p2112 peptide in solution observed by NMR. Overlay of the 1H-15N TROSY spectra of free PCNA (black) and PCNA bound to the p2112 (red) peptide. These two spectra were measured at 800 MHZ and 35°C on a 125 µM PCNA sample in PBS pH 7.4, with a 1∶11 PCNA:p21 peptide ratio, on a monomer basis. (TIF) [file pone.0048390.s004.tif]

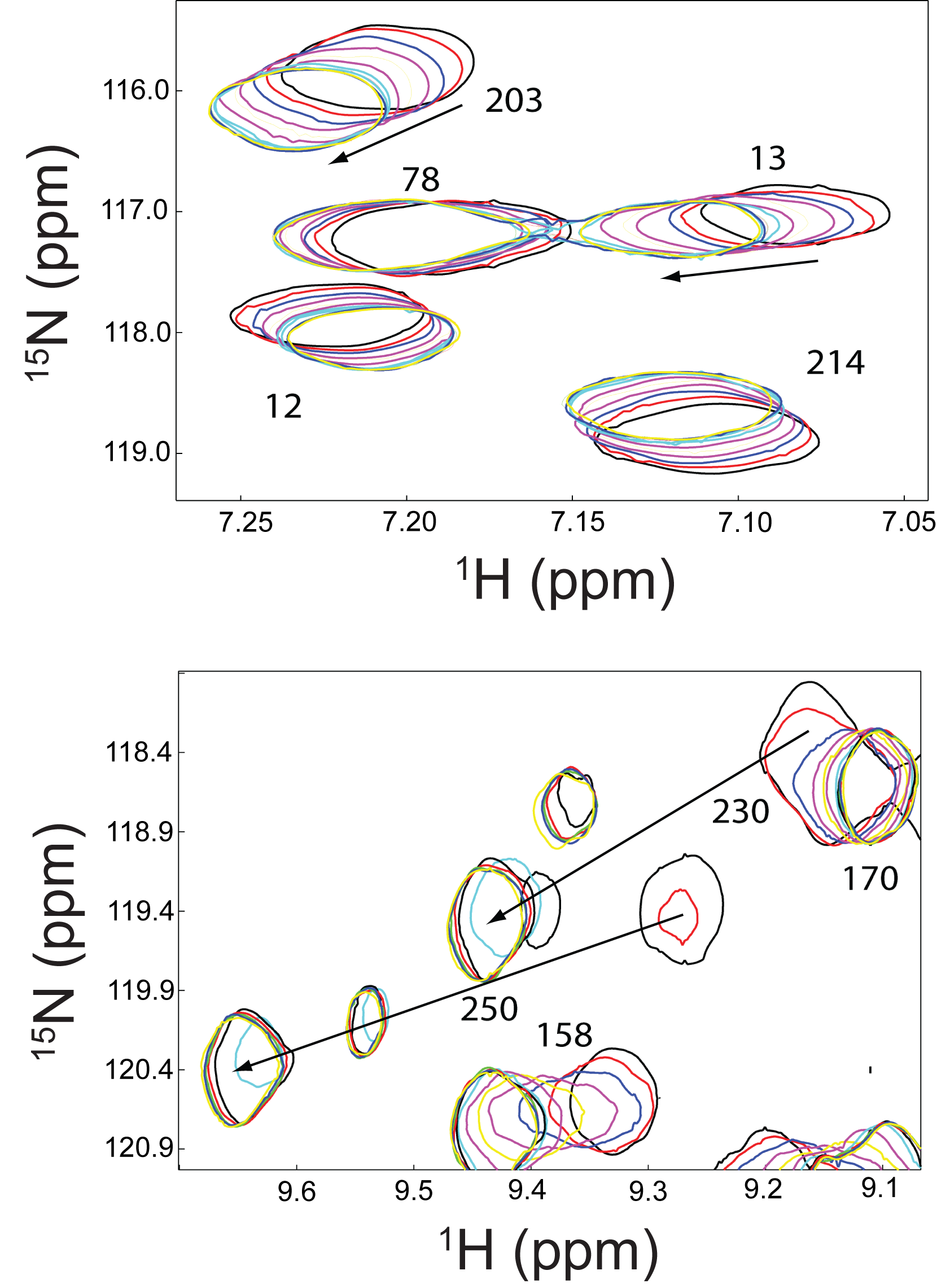

Supplement: Figure S5 — Titration of PCNA with the p2112 peptide. The two panels show representative regions of thirteen superimposed 1H-15N HSQC spectra of 0.9 mM of uniformly labeled 2H,13C,15N-PCNA in the presence of increasing concentrations of p2112 peptide (the different colors correspond to peptide concentration values from 0 to 1.3 mM in 0.1 mM increments). The arrows indicate the signal movement during the titration for selected amide resonances: residues 12, 13, 78, 203 and 214 (top panel) are in fast exchange, residue 158 (bottom panel) in intermediate exchange, and residues 230 and 250 (right panel) in slow exchange. (TIF) [file pone.0048390.s005.tif]

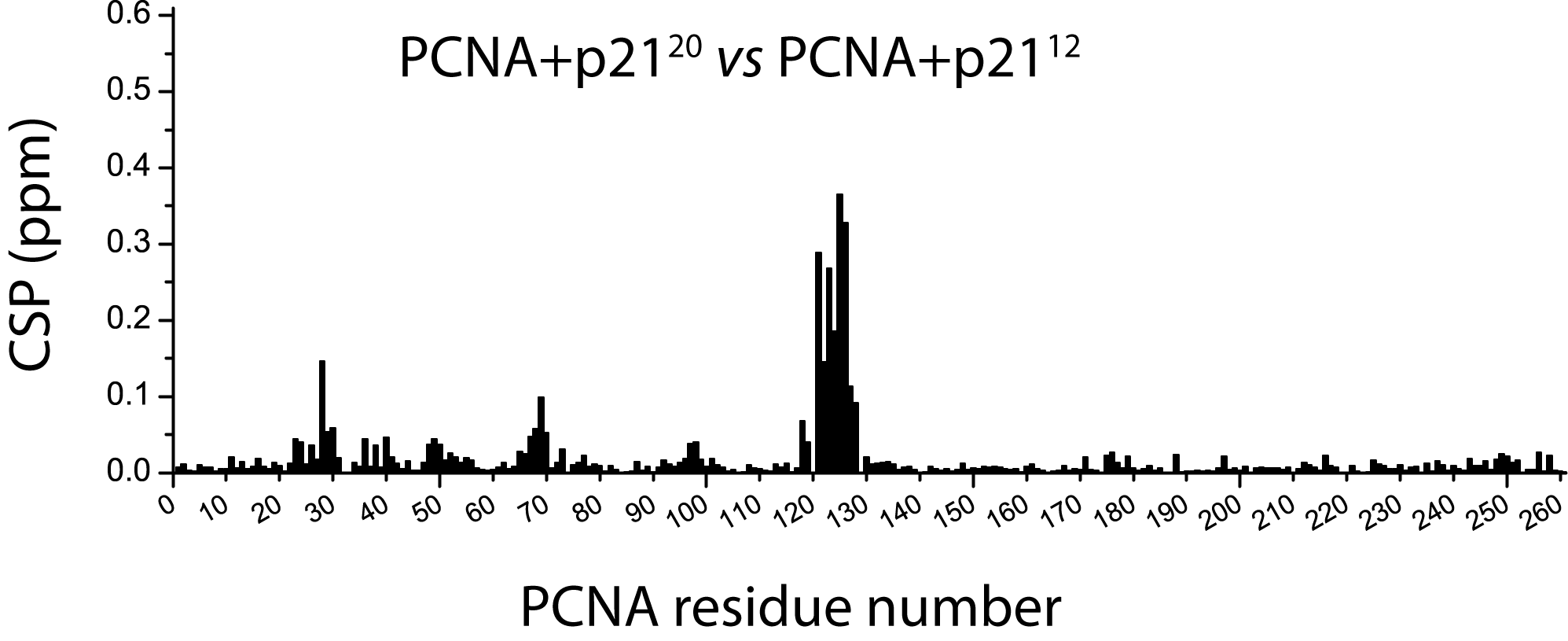

Supplement: Figure S6 — Differences in the CSP of PCNA bound to p2120 or to p2112 peptide. These CSP values were calculated from the chemical shifts measured on spectra of PCNA bound to p2120 and using as reference the chemical shifts of PCNA bound to p2112. (TIF) [file pone.0048390.s006.tif]

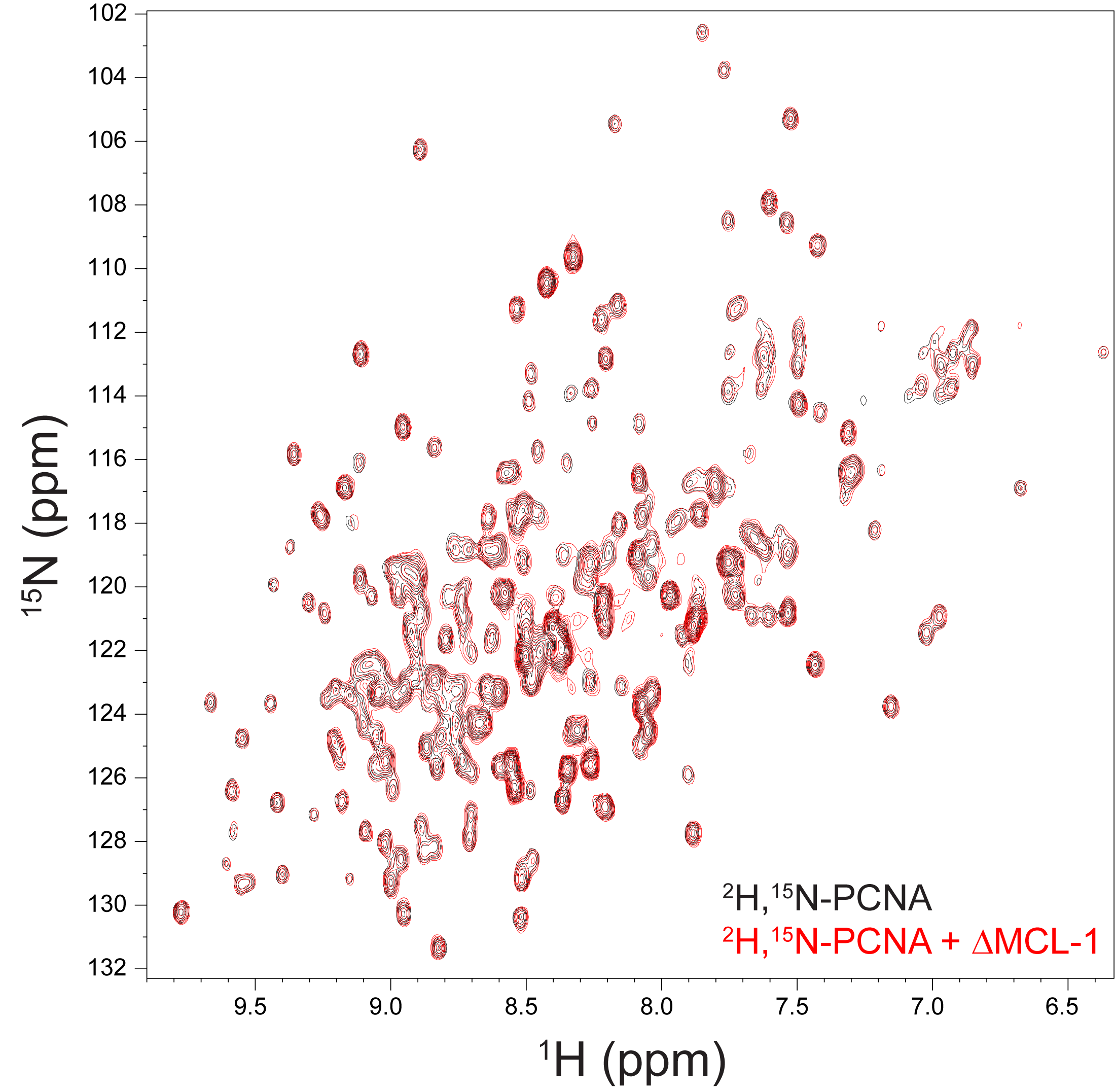

Supplement: Figure S7 — NMR examination of the interaction of PCNA with MCL-1. 1H-15N TROSY spectra of 50 µM uniformly labeled 2H-15N PCNA alone (black) or in the presence (red) of 220 µM of the ΔN151−ΔC7 fragment of human MCL-1 (1∶4.4 ratio on a PCNA monomer basis). The spectra were recorded in 10 mM Tris pH 7.4, 250 mM NaCl, 1 mM EDTA, 0.5 mM DTT at 800 MHz and 35°C. The weak red signals around 8.3 and 122 ppm in the 1H and 15N chemical shift dimensions, respectively, arise from degradation products of PCNA. This interpretation is based on the observation that their chemical shifts are typical of random coil polypeptides, and that their number and intensity increase over time. This interpretation was confirmed by SDS-PAGE and mass spectrometry analysis of the NMR sample. We think that PCNA proteolysis is caused by traces of proteases that co-purified with the MCL-1 protein. This experiment was repeated using a different batch of purified MCL-1 protein with the same results. A small systematic reduction in the intensity of the PCNA signals was observed in the spectrum of the mixture relative to PCNA alone, which can be explained by contributions from i) the decrease in the concentration of intact PCNA protein over time due to proteolysis, ii) the increase in the medium viscosity causing a slower tumbling of the protein, and iii) a possible non-specific binding into large aggregates that are not visible by NMR. Similar observations were made on samples with different molar ratios of the two proteins and a with a shorter MCL-1 fragment (named the core domain of MCL-1, cMCL-1 or ΔN162−ΔC24) which contains residues 162–327 (data not shown). If MCL-1 were specifically bound to PCNA, a localized, non-uniform signal intensity reduction, in addition to chemical shift perturbations, should be observed in the PCNA signals. (TIF) [file pone.0048390.s007.tif]

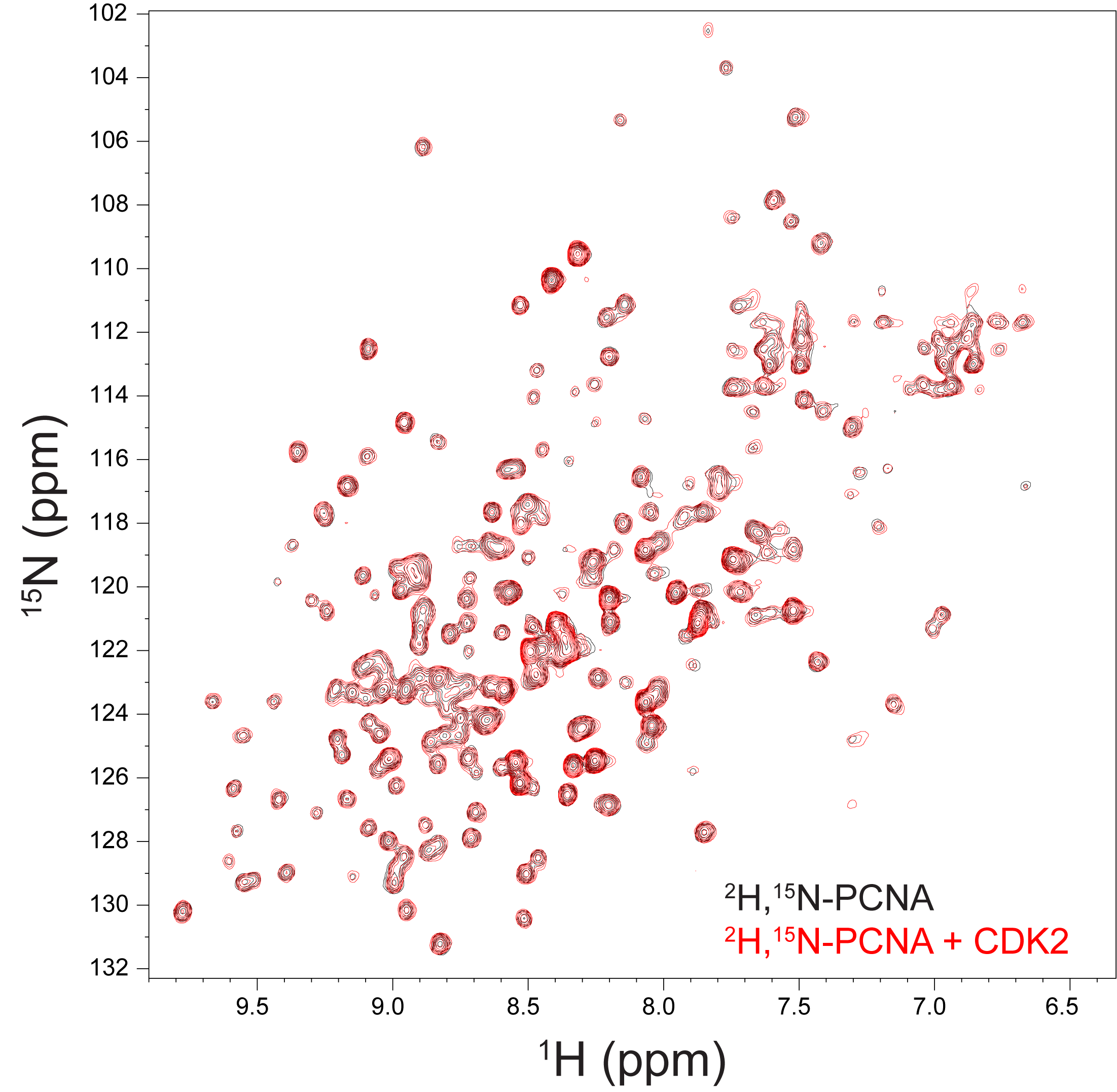

Supplement: Figure S8 — NMR examination of the interaction of PCNA with CDK2. 1H-15N TROSY spectra of uniformly labeled 50 µM 2H-15N PCNA alone (black) or in the presence (red) of CDK2 (1∶1 ratio on a PCNA monomer basis). The spectra were recorded in 20 mM Tris pH 7.2, 150 mM NaCl, 1 mM DTT at 800 MHz and 35°C. (TIF) [file pone.0048390.s008.tif]
