# Supplementary figures and images for: Correction: Proliferating Cell Nuclear Antigen (PCNA) Interactions in Solution Studied by NMR
Source: PLoS One. 2014 Apr 18;9(4):e95818. doi: 10.1371/journal.pone.0095818 (PMC3991720; doi:10.1371/journal.pone.0095818)

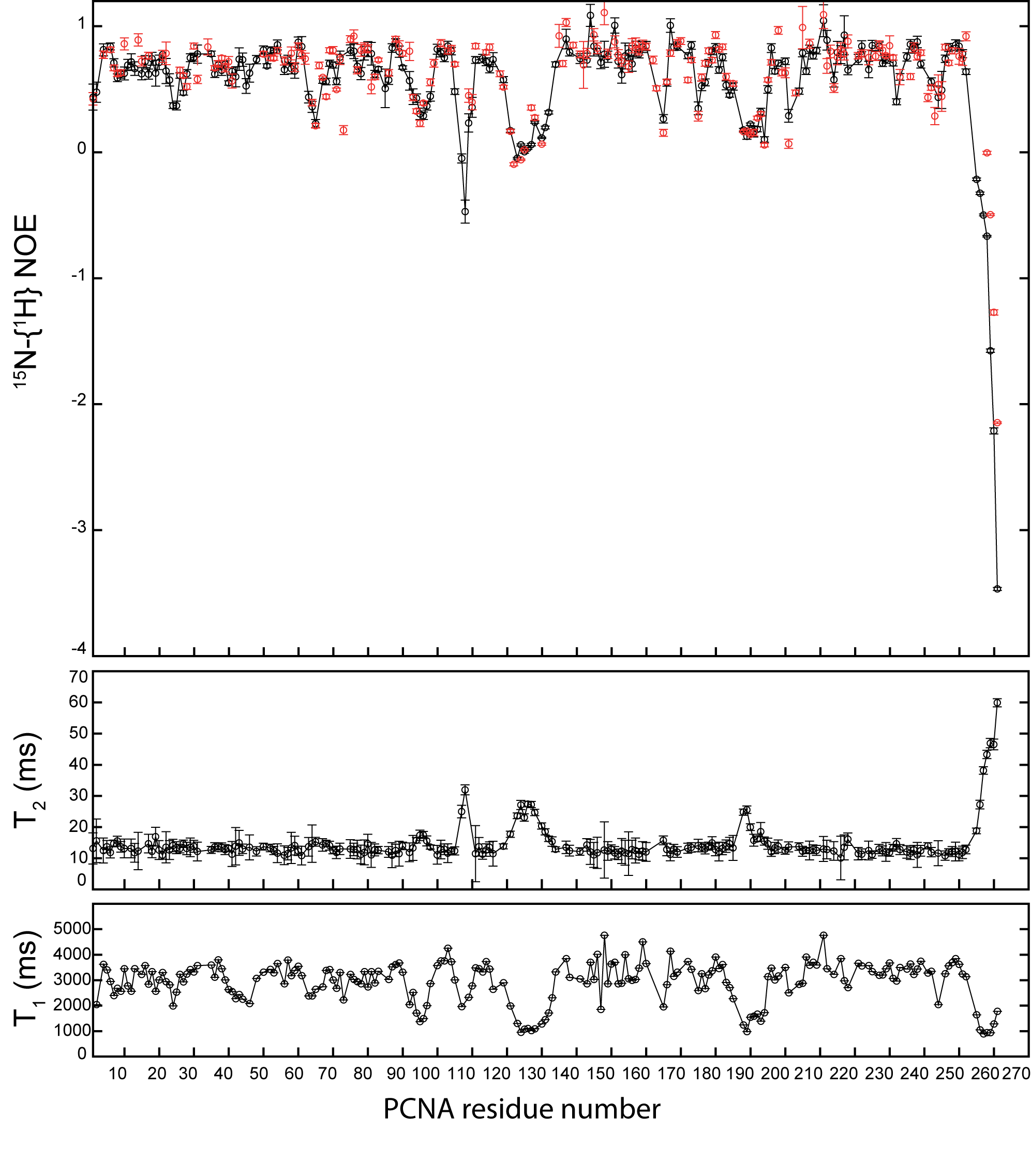

Supplement: Figure S1 — Changes in PCNA backbone dynamics upon p2112 binding. Backbone amide 15N NMR relaxation parameters for PCNA at 60 MHz in PBS pH 7.0 at 35°C. The heteronuclear {1H}-15N NOEs, and 15N transversal (T2) and longitudinal (T1) relaxation times are represented for each residue of PCNA in its free form (black open circles) and, in the case of the {1H}-15N NOEs, also bound to p2112 peptide (red open circles). (TIF) [file pone.0095818.s001.tif]

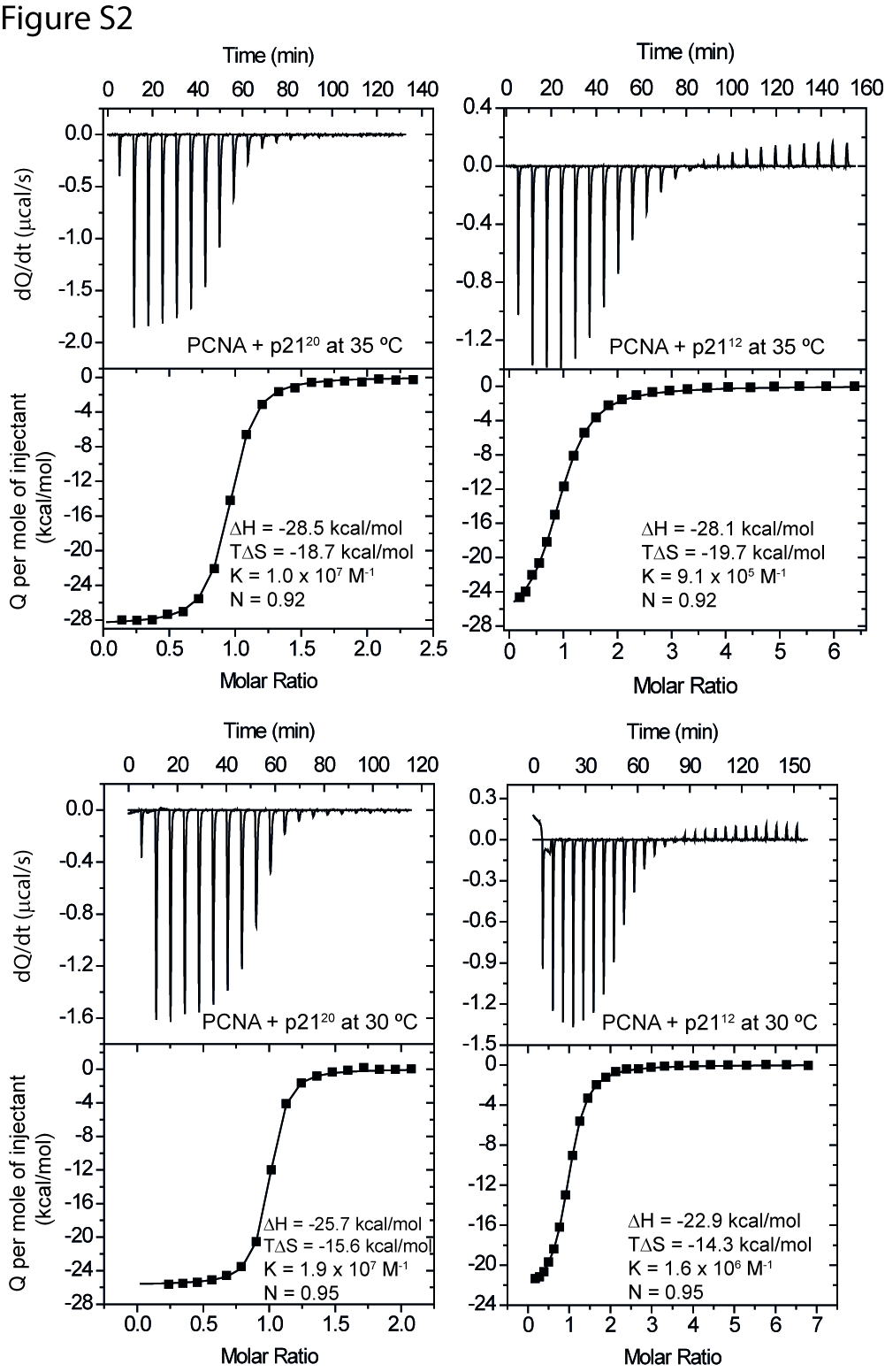

Supplement: Figure S2 — Calorimetric titrations of PCNA with p21 peptides at 30 and 35°C. For each graph the upper panels represent the heat effect associated with the peptide injections and the lower panels represent the ligand concentration dependence of the heat released upon binding, after normalization and correction for the heats of dilution. In the lower panels the symbols are the experimental data, and the continuous line is the best fit to a model of one set of identical binding sites. (TIF) [file pone.0095818.s002.tif]
